# Imbalanced functional link between executive control network and reward network explain the online-game seeking behaviors in Internet gaming disorder

**DOI:** 10.1038/srep09197

**Published:** 2015-03-17

**Authors:** Guangheng Dong, Xiao Lin, Yanbo Hu, Chunming Xie, Xiaoxia Du

**Affiliations:** 1 Department of Psychology, Zhejiang Normal University, Jinhua, Zhejiang Province, PR China; 2 Centre for Integrative Neuroscience and Neurodynamics, School of Psychology and Clinical Language Sciences, University of Reading, U.K.; 3 Medical School of Southeast University, Nanjing 210009, PR China; 4 Department of Physics, Shanghai Key Laboratory of Magnetic Resonance, East China Normal University, Shanghai, PR China

## Abstract

Literatures have shown that Internet gaming disorder (IGD) subjects show impaired executive control and enhanced reward sensitivities than healthy controls. However, how these two networks jointly affect the valuation process and drive IGD subjects' online-game-seeking behaviors remains unknown. Thirty-five IGD and 36 healthy controls underwent a resting-states scan in the MRI scanner. Functional connectivity (FC) was examined within control and reward network seeds regions, respectively. Nucleus accumbens (NAcc) was selected as the node to find the interactions between these two networks. IGD subjects show decreased FC in the executive control network and increased FC in the reward network when comparing with the healthy controls. When examining the correlations between the NAcc and the executive control/reward networks, the link between the NAcc - executive control network is negatively related with the link between NAcc - reward network. The changes (decrease/increase) in IGD subjects' brain synchrony in control/reward networks suggest the inefficient/overly processing within neural circuitry underlying these processes. The inverse proportion between control network and reward network in IGD suggest that impairments in executive control lead to inefficient inhibition of enhanced cravings to excessive online game playing. This might shed light on the mechanistic understanding of IGD.

Unlike drug addictions or substance abuse, internet gaming disorder (IGD) has no chemical or substance intake while still leads to physical dependence, similar to other addictions^1^,^2^. People's online experience may change their cognitive function in a manner that drive their online game playing, which also occurs in the absence of drug taking^1^,^3^,^4^. The DSM-5 considering substance-use disorders and addictions generated criteria for Internet gaming disorder, and this disorder is included in the section of the DSM-5 containing disorders warranting additional study^5^,^6^. At the neural system level, however, the precise mechanisms underlying the cognitive control failure are far from clear^7^.

One key feature of IGD is the loss of volition to control online-game seeking behaviors. Recent functional magnetic resonance imaging (fMRI) studies identified two important neuronal activity patterns in IGD: First, reduced response inhibitions were demonstrated in the IGD subjects using go/no-go^8^, task switching^9^,^10^, and the Stroop^11^,^12^,^13^ tasks compared with healthy controls (HC); Second, IGD subjects showed enhanced reward sensitivity than HC^2^,^14^,^15^ and showed cognitive bias toward information derived from the Internet^9^,^16^,^17^. These two features are much similar to the findings from current neuro-economic studies– There are two distinct brain networks that jointly influence decision-making processes^18^,^19^: The executive control network (involves the lateral prefrontal and parietal cortices^19^), which is related to delayed rewards; The ventral valuation network (involves the orbitofrontal cortex, ventral striatum and so on^19^,^20^), mediates for immediate rewards.

The interactions between these two networks are also demonstrated in drug addicted groups^20^. Xie's study showed an imbalanced functional link between control network (decreased links) and reward network (enhanced links) in Heroin-dependent subjects^21^, which can shed light on the mechanistic understanding of drug addiction at a large-scale system level. The enhanced motivations to seek drugs combined with an inability to inhibit drug-related behaviors are thought to represent a failure of executive control^22^,^23^,^24^. In studies with IGD, researchers have observed similar features in the executive control and the reward sensitivity (as mentioned before). However, how these two networks jointly affect the valuation process in IGD subjects and drive their online-game-seeking behaviors remains unknown.

Recently, studies have investigated the neural activities in the human brain during resting state (no stimuli, no tasks, not fall asleep), which termed resting-states fMRI. They found that the neural activities during resting state are correlated across cortical regions with specific functional properties, but not random^25^,^26^,^27^. These temporal correlations are presumed to reflect intrinsic functional connectivity (FC) and have been demonstrated across several distinct networks^28^,^29^,^30^. It can be a useful tool to investigate the potential neuronal network differences at a more intrinsic level between the IGD and the HC groups during resting state.

The temporal binding model suggests that the synchronization of brain signals between neural systems is crucial in facilitating neural communications^31^. Literatures have also proved that the resting FC can be a predictor of behavioral performance^26^,^32^. As we mentioned above, the IGD subjects showed decreased executive control and increased reward sensitivity than the HC. We hypothesize that IGD subjects show enhanced synchrony in reward network and decreased synchrony in control network than HC. In addition, we also hypothesize that the underlying duality of the control/reward networks that jointly influence valuation was impaired in IGD. To test these hypotheses, we first need to measure the resting-states fMRI; Second, we need to select some seeds to represent different networks and measure these seed-based BOLD signals, which is to establish the links between these two networks; Third, we need to measure their interactions to find how they jointly work on behaviors.

## Methods

### Participant selection

The experiment conforms to The Code of Ethics of the World Medical Association (Declaration of Helsinki). The Human Investigations Committee of Zhejiang Normal University approved this research. The methods were carried out in accordance with the approved guidelines. Participants were university students and were recruited through advertisements. Participants were right-handed males (35 IGA subjects, 36 healthy controls (HC)). IGD and HC groups did not significantly differ in age (IGA mean = 22.21, SD = 3.08 years; HC mean = 22.81, SD = 2.36 years; *t* = 0.69, *p* = 0.49). Only males were included due to higher IGD prevalence in men than women. All participants provided written informed consent and a structured psychiatric interviews (M.I.N.I.)^33^ that performed by an experienced psychiatrist, which need approximately 15 minutes. All participants were free of Axis I psychiatric disorders listed in M.I.N.I. We further assessed ‘depression' with the Beck Depression Inventory^34^ and only participants scoring less than 5 were included. All participants were instructed not to use any substances of abuse, including caffeine drinks, on the day of scanning. No participants reported previous usage of illicit drugs (e.g., cocaine, marijuana).

Internet addiction disorder was determined based on Young's online internet addiction test (IAT)^35^ scores of 50 or higher. Young's IAT consists of 20 items from different perspectives of online internet use, including psychological dependence, compulsive use, withdrawal, problems in school or work, sleep, family or time management^35^. The IAT was proved to be a valid and reliable instrument that can be used in classifying IAD^36^,^37^. For each item, a graded response is selected from 1 = “Rarely” to 5 = “Always”, or “Does not Apply”. Scores over 50 indicate occasional or frequent internet-related problems) (www.netaddiction.com). When selecting IGD subjects, we added an addition criterion on Young's established measures of IAT: ‘you spend ___% of your online time playing online games' (>80%).

### Scanning of resting-states data

The scan was performed in the MRI center in East-China Normal University. MRI data were acquired using a Siemens Trio 3T scanner (Siemens, Erlangen, Germany). The ‘resting state' was defined as no specific cognitive task during the fMRI scan in our task. Participants were required to keep still, close their eyes, remain awake and not to think of anything systematically^38^,^39^. To minimize head movement, participants are lain supine with head snugly fixed by belt and foam pads. The resting-state functional images were acquired by using an EPI (echo-planar imaging) sequence. The scan parameters are as following: interleaved, repetition time = 2000 ms, 33 axial slices, thickness = 3.0 mm, in-plane resolution = 64* 64, echo time = 30 ms, flip angle = 90, field of view = 240* 240 mm, 210 volumes (7 min). Structural images were collected using a T1-weighted 3D spoiled gradient-recalled sequence, and was acquired covering the whole brain (176 slices, repetition time = 1700 ms, echo time TE = 2.26 ms, slice thickness = 1.0 mm, skip = 0 mm, flip angle = 90°, field of view = 240*240 mm, in-plane resolution = 256* 256).

### Data pre-processing

The resting data was performed using REST and DPARSF (http://restfmri.org)^40^. Preprocessing consisted of removal of the first 10 time points (due to signal equilibrium and to allow the participants to adapt to the scanning noise), physiological correction, slice timing, volume registration and head motion correction. Possible contamination from several nuisance signals including the signal of white matter, cerebral spinal fluid, global signal, and six motion vectors were regressed out. The time series of images of each subject were motion-corrected using a least squares approach and a six-parameter (rigid body) linear transformation^41^. The individual structural image was co-registered to the mean functional image after motion correction using a linear transformation. The motion corrected functional volumes were spatially normalized to the MNI (Montreal Neurological Institute) space and re-sampled to 3-mm isotropic voxels using the normalization parameters estimated during unified segmentation. Further preprocessing include (1) band-pass filtering between 0.01 and 0.08 Hz; (2) To assess functional connectivity, we first calculated the Pearson's correlation coefficient between the mean signal intensity time courses of each region of interest (ROI) pair. A Fisher's r-to-z transformation was applied to each correlation map to obtain an approximately normal distribution of the functional connectivity values and to accordingly apply parametric statistics.

### ROI selection in rest

**Figure 1:**
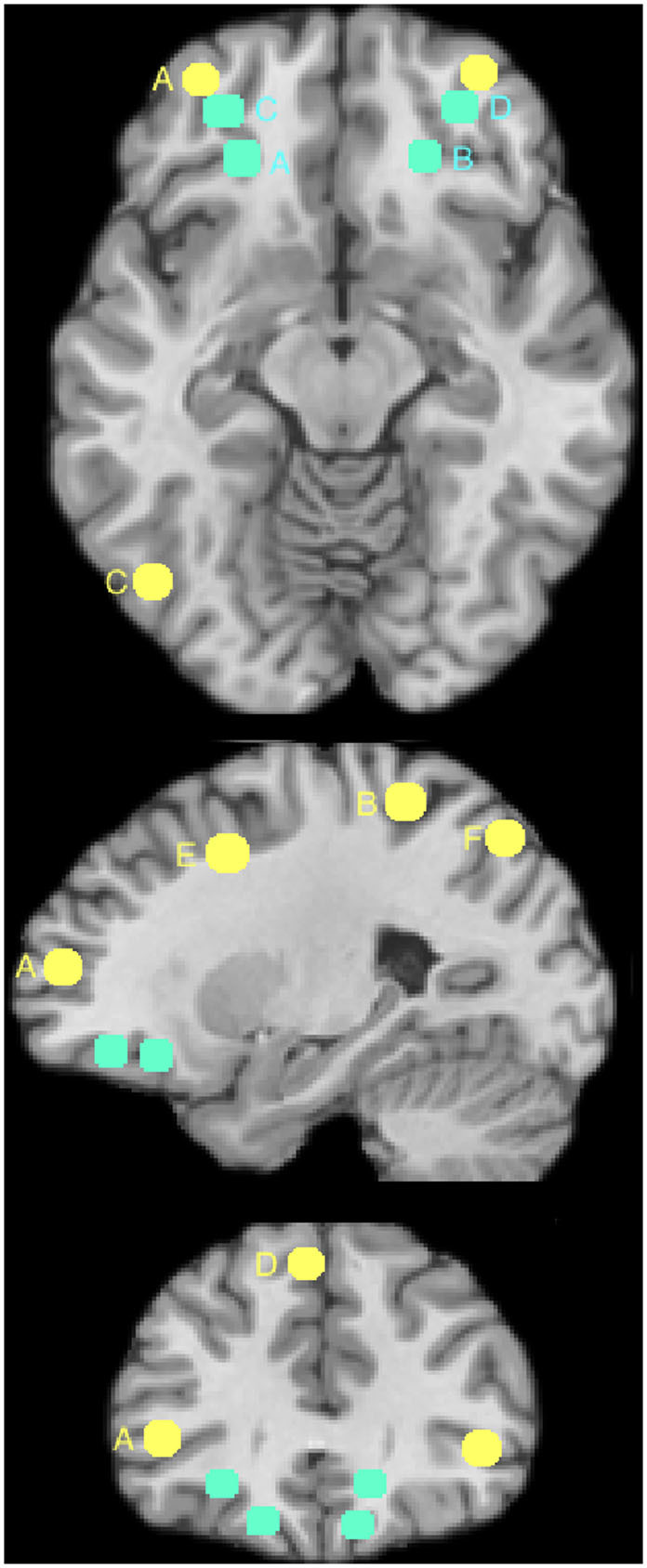
The ROIs selected in the research. **Yellow**: The control network. Six control ROIs in left hemisphere. This figure and ROIs were extracted from Yeo et al. (2011)^42^, please detailed information and exact coordinates from that paper. The coordinates of these six ROIs: A: −40, 50, 7; B: −43, −50, 46; C: −57, −54, −9; D: −5, 22, 47; E: −6, 4, 29; F: −4, −76, 45; radius = 6 mm). **Blue**: The OFC seeds selected as part ROIs of the reward network in this study. Please find detailed information and exact coordinates from Kringelbach et. al. (2004)^44^. The coordinates of these four ROIs: A: −23, 30, −12; B: 16, 29, −13; C: −32, 40, −11; D: 33, 39, −11. The other reward network seeds include striatum and amygdala. The current figure is just to show their approximate locations. It is really hard to label all ROIs in one X-Y-Z figure. Please find their exactly locations from the original publications.

Seeds were chosen as priori based on published literatures rather than deriving seed regions from tasks is to avoid bias and to increase the generalizability of findings. For the control network, seeds were defined based on a recent FC study using data from 1000 young adults^42^ suggesting frontal-parietal control network includes six brain regions. They located in frontal and parietal area of the brain (find detailed coordinates from Figure 1). We used the symmetric coordinates to select the seeds from the right hemisphere.

For reward valuation network, plenty of studies have suggested that the orbitofrontal striatal circuit support the conversion of disparate types of future rewards into a kind of internal currency^18^,^20^,^21^. This circuit include ventral striatum, dorsal striatum, and orbitofrontal circuit. Besides this, previous studies also showed that the amygdala network is the key region that underlying reward valuation^43^. Thus, in this study, we also included amygdala into the reward network. Because the striatum, amygdala are relative small brain regions, we selected the whole region as seeds. The amygdala was extracted from Harvard-Oxford subcortical atlas; the striatum were selected using Oxford-striatum-atlas. For the OFC, seeds were defined based on a meta-analysis^44^,^45^, which suggesting two distinct lateral OFC functional sub-regions, one involved in motivation-independent reinforcer representations (−23, 30, −12 and 16, 29, −13) and another in evaluation of punishers leading to change in behaviour (−32, 40, −11 and 33, 39, −11). See Figure 1.

The connections between seeds we selected above can only provide the group-level differences and show the inner-connections inside control network and reward network, separately. To find the interactions between these two networks for individual subjects and how they jointly influence the behaviors, we need a “node” that connects to both the networks. In this study, we selected the nucleus accumbens (NAcc) region as a connective node or a ‘seed' region to link between the control and reward networks because the NAcc has an important role in addiction^46^, and were proved to be a valuable connective node in addiction studies^21^. The NAcc were also extracted from Harvard-Oxford subcortical atlas.

### Functional Connectivity calculation

For each ROI, a representative BOLD time course was obtained by averaging the signal of all the voxels within the ROI. Literatures on functional networks have shown to have separable right and left hemisphere components^47^,^48^,^49^. Thus, in this study, we first calculated the mean value of FCs among left and right control/reward network ROIs, separately. Then, we took the mean value of these two FCs as the whole FC index. The correlation between NAcc and executive/reward network was calculated as follows: We calculated the mean value of FCs between NAcc and control/reward network ROIs in same hemisphere. Then, we took the mean value of these hemispheric FCs as the overall FC index.

## Results

### FC difference in control network between IGD and HC

**Figure 2:**
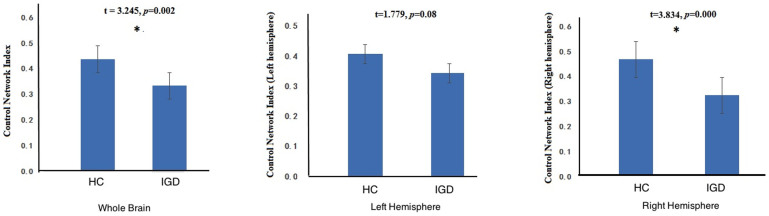
Composite FC indices of control network in IGD and HC groups in different comparisons: the whole brain (left), left hemisphere (middle), and right hemisphere (right).

Figure 2 shows the FC in control network in IGD and HC. The FC in control network in HC is significant higher than that in IGD, at both the whole brain and the hemispheric levels (HC is marginally significant than IGD in the FC in left control network).

### FC difference in reward network between IGD and HC

**Figure 3:**
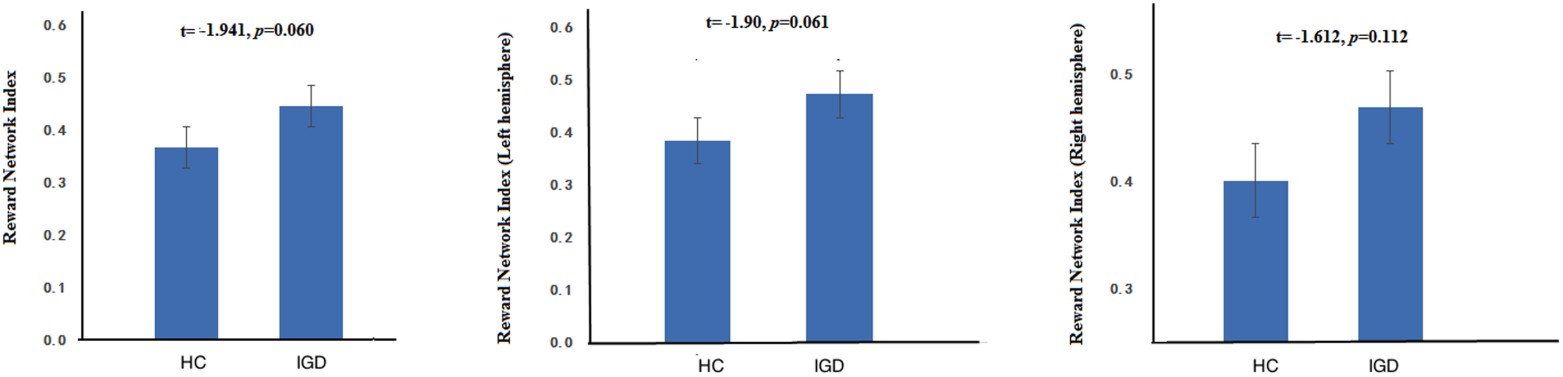
Composite FC indices of reward network in IGD and HC groups in different comparisons: the whole brain (left), left hemisphere (middle), and right hemisphere (right).

Figure 3 shows the FC in reward network in IGD and HC. The FC in IGD reward network is marginally significant higher than that in HC in whole brain (*p* = 0.060) and left hemisphere (*p* = 0.061). Although IGD show higher FC than HC in right hemisphere, however, it does not reach statistical significance (*p* = 0.112).

### Interactions between control network and reward network

**Figure 4:**
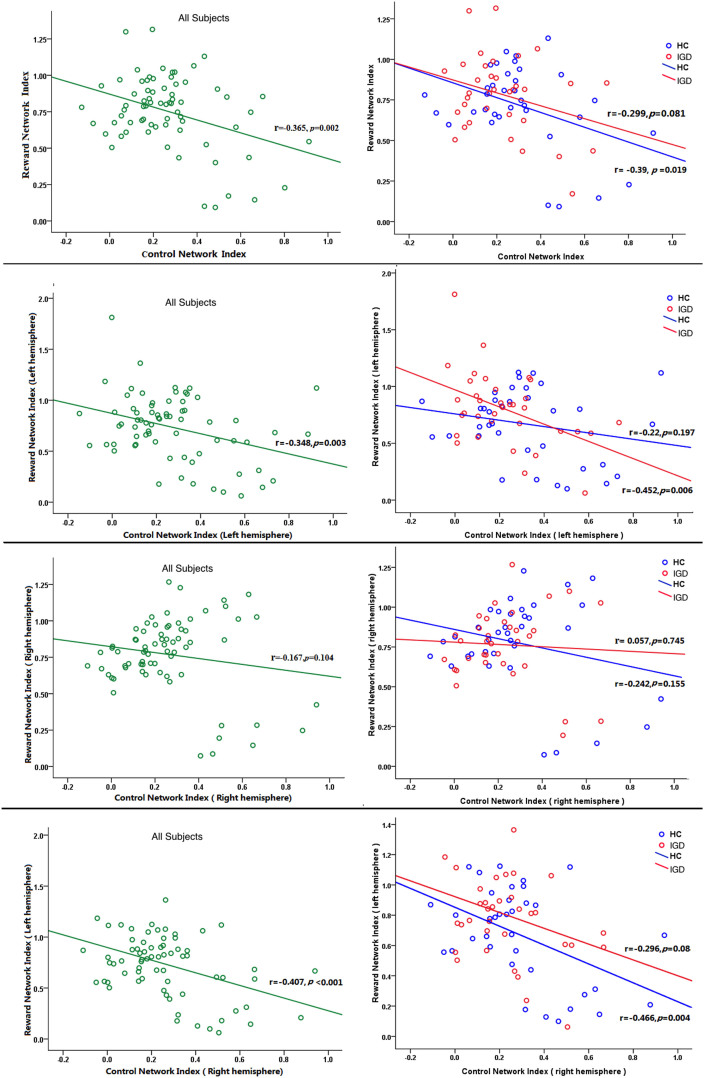
The relationship between control network and reward network indices in all subjects (left), IGD (middle) and HC groups (right), respectively. Different rows show different comparisons: the whole brain (first row), left hemisphere (second row), right hemisphere (third row), and between-hemispheres (fourth row).

We calculated the interactions between control network and reward network in whole brain level and hemispheric levels. The first row of figure 4 shows the relation between control network and reward network in whole brain in all subjects (left), and in groups (right). We can find the FC in control network is negatively correlated with reward network in all groups of subjects. The figures in the second row show that control network is inversely correlated with reward network in left hemisphere. However, in right hemisphere (the third row), although they show negative trends, all of these correlations do not reach statistical significance (This might because all the control network ROIs were defined in left hemisphere. The ROIs in right hemisphere were selected according to left hemisphere symmetrically). The fourth row showed the between-hemispheric interactions between control network and reward network. We can also find the negative correlation between control network and reward network. Take all, although a few of these correlations do not reach statistical significance, we can still infer that control network is negatively related with reward network.

## Discussion

### Lower control network synchrony and higher reward network synchrony in IGD subjects

In this study, we observed decreased synchrony of the executive control network of IGD subjects comparing to that of HC. The temporal binding model suggests that the synchronization of brain signals between brain regions is crucial in facilitating neural communications^31^. Thus, the decreased synchrony in control network might indicate that IGD subjects' long time online-game playing impaired their executive control system. Previous studies have found that the FC in specific network can be a predictor of relevant behavioral performance^30^,^50^,^51^. Task based fMRI studies also demonstrated that IGD subjects showed reduced response inhibitions than healthy controls^8^,^9^,^11^,^12^. Such response tendencies appear to be influenced by online-gaming related stimuli, with worse performance seen in IGD than in non-IGD subjects^9^. Apparent set-shifting and cognitive control deficits in IGD may be related to the inefficient processing within neural circuitry underlying these processes, with some of these neural measures relating to IGD severity^12^.

In the reward network, the FC in IGD is marginally significant higher than that in HC. The stronger links among reward network seeds in IGD suggested that they showed enhanced reward craving to reward than HC group. Task based fMRI studies have shown evidences that the reward sensitivity is elevated among IGD subjects when comparing to healthy controls^2^,^9^,^14^,^15^ in both mild and extreme situations. The enhanced reward sensitivity may contribute to the increased desires to engage in online game playing, due to the IGD subjects may experience stronger reward. And the long-term online gaming may lead players to indulge in virtual experiences and relive these experience in real life^52^.

### Imbalanced correlation between control network and reward network

To further test the interactions between the executive control network and the reward network and to find how they jointly influence the final behaviors in individual subjects, we selected the NAcc as a connective node or a ‘seed' region to link the executive control and the reward networks. Figure 4 shows that the indices of the executive control network and the reward network have a significant inverse proportions, which suggests the stronger the reward network connectivity, the weaker the control network connectivity. These two network interact in a pull and push fashion where strong motivation will lead to the disturbance of the executive control circuit, and the strong executive control will lead to the inhibition of the motivational desires^53^.

Previous studies have demonstrated that the executive control system promotes cognitive and behavioral control over motivational drives and may enable individuals to inhibit desires and reward-seeking behaviors^54^,^55^,^56^. The inverse proportion between the executive control network and the reward network might contribute a lot in understanding the addictive mechanism underlying IGD: Increased reward sensations during winning or pleasurable experience may enhance their desire to play online. Meanwhile, impairments in executive control may lead to inefficient inhibition of such desires, which may permit urges, desires or cravings to dominate and lead to excessive online game playing.

The imbalanced functional link between the executive control network and the reward network may also shed light on the understanding of IGD's decision making. Studies revealed that IGD subjects show diminished consideration of experiential outcomes when making future decisions^52^. In making decisions between participating in immediately rewarding experiences (e.g., playing online) and long-term adverse consequences (e.g., using the time spent gaming instead to perform activities associated with longer term occupational success), individuals with IGD may be considered as showing a “myopia for the future”, as has been described for drug addictions^57^,^58^,^59^. The strong reward network synchrony of immediate reward might overdrives the decision process to inhibit the impulse, which might be reasonable to explain the valuation-based decision-making process toward the immediate reward, resulting in the impulsive online-game playing behaviors. In addition, reward-seeking behaviors may be reinforced through short-term online experiences, leading to a vicious cycle of addictive online game playing^7^.

To sum up, this study showed that the changes (decrease/increase) in IGD subjects' brain networks synchrony suggest the inefficient/overly processing within neural circuitry underlying these processes. The inverse proportion between the executive control network and the reward network suggest that impairments in the executive control lead to inefficient inhibition of enhanced cravings to excessive online game playing. These results might shed light on the mechanistic understanding of IGD. In addition, the similar features between IGD and drug addictions (for example, Heroin dependence) suggest IGD may share the similar neural underpinnings with other types of addictions.

### Limitations

Several limitations should be addressed here. First, because there are only few females addicted to online games, we only selected male subjects in this study. The imbalance in gender might limit the final conclusions. Second, in calculating the interactions between control networks and reward networks, we selected the NAcc as the seed based on the functionality of the NAcc and previous literatures. We don't know whether there are better seeds for this calculation. Third, the present study only revealed the current states existed in IAD subjects, we cannot draw causal conclusions between these factors. Fourth, in selecting the right hemisphere ROIs for the executive control network, we used the symmetric coordinates according to the left hemisphere, which might be the reason why the indexes in right hemisphere are lower than that in the left hemisphere.

## Author Contributions

G.D. designed the experiment and wrote the first draft of the manuscript. X.L. and X.D. collected and analyzed the data, prepared the figures. Y.H. and C.X. discussed the results, advised on interpretation and contributed to the final draft of the manuscript. All authors contributed to and have approved the final manuscript.
